# Changing flood frequencies under opposing late Pleistocene eastern Mediterranean climates

**DOI:** 10.1038/s41598-018-25969-6

**Published:** 2018-05-31

**Authors:** Yoav Ben Dor, Moshe Armon, Marieke Ahlborn, Efrat Morin, Yigal Erel, Achim Brauer, Markus Julius Schwab, Rik Tjallingii, Yehouda Enzel

**Affiliations:** 1 The Fredy and Nadine Herrmann Institute of Earth Sciences, The Hebrew University of Jerusalem, Jerusalem, Israel; 2 Section 5.2: Climate Dynamics and Landscape Evolution, GFZ German Research Centre for Geosciences, Potsdam, Germany

## Abstract

Floods comprise a dominant hydroclimatic phenomenon in aridlands with significant implications for humans, infrastructure, and landscape evolution worldwide. The study of short-term hydroclimatic variability, such as floods, and its forecasting for episodes of changing climate therefore poses a dominant challenge for the scientific community, and predominantly relies on modeling. Testing the capabilities of climate models to properly describe past and forecast future short-term hydroclimatic phenomena such as floods requires verification against suitable geological archives. However, determining flood frequency during changing climate is rarely achieved, because modern and paleoflood records, especially in arid regions, are often too short or discontinuous. Thus, coeval independent climate reconstructions and paleoflood records are required to further understand the impact of climate change on flood generation. Dead Sea lake levels reflect the mean centennial-millennial hydrological budget in the eastern Mediterranean. In contrast, floods in the large watersheds draining directly into the Dead Sea, are linked to short-term synoptic circulation patterns reflecting hydroclimatic variability. These two very different records are combined in this study to resolve flood frequency during opposing mean climates. Two 700-year-long, seasonally-resolved flood time series constructed from late Pleistocene Dead Sea varved sediments, coeval with significant Dead Sea lake level variations are reported. These series demonstrate that episodes of rising lake levels are characterized by higher frequency of floods, shorter intervals between years of multiple floods, and asignificantly larger number of years that experienced multiple floods. In addition, floods cluster into intervals of intense flooding, characterized by 75% and 20% increased frequency above their respective background frequencies during rising and falling lake-levels, respectively. Mean centennial precipitation in the eastern Mediterranean is therefore coupled with drastic changes in flood frequencies. These drastic changes in flood frequencies are linked to changes in the track, depth, and frequency of mid-latitude eastern Mediterranean cyclones, determining mean climatology resulting in wetter and drier regional climatic episodes.

## Introduction

**Figure 1:**
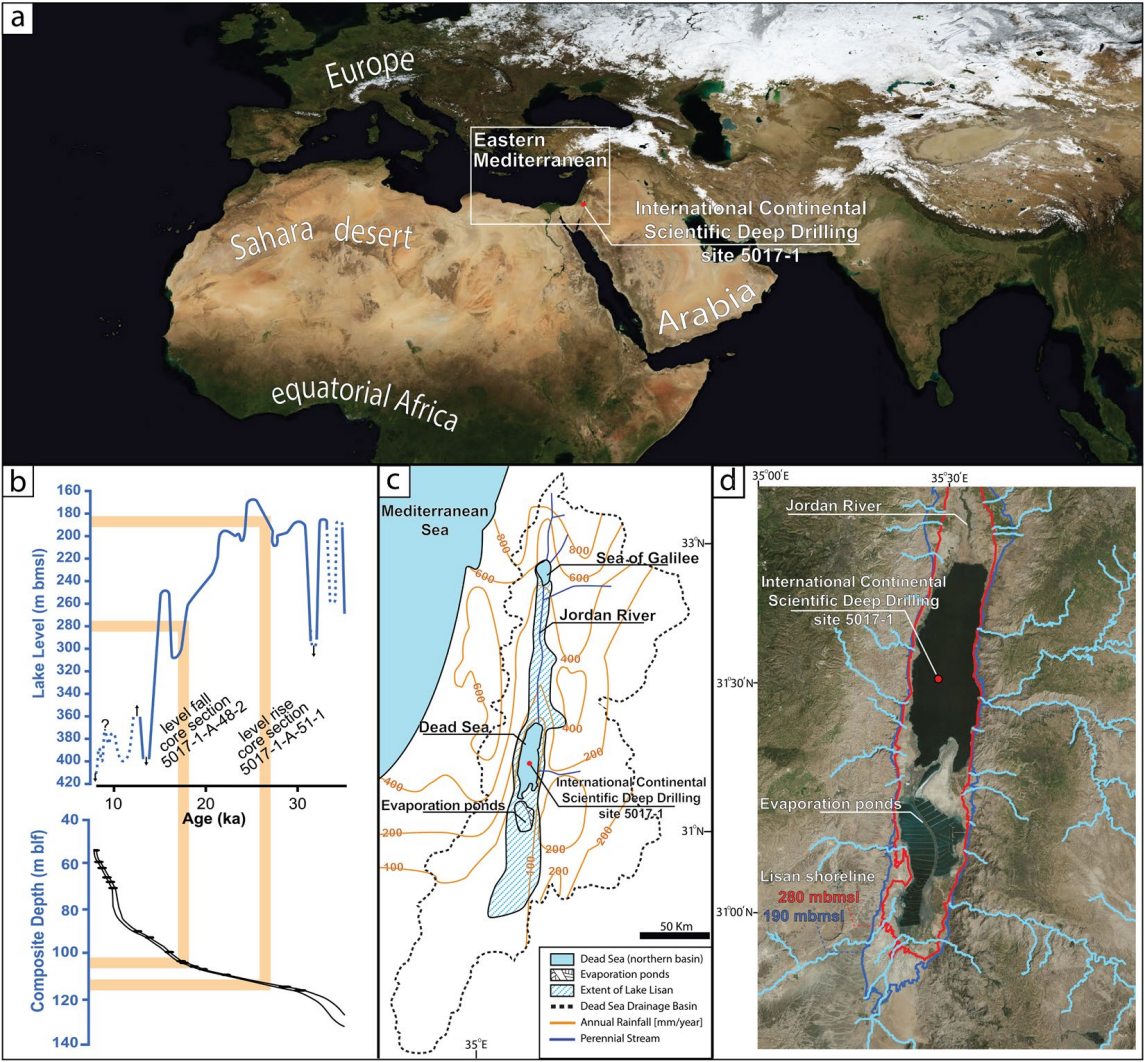
(**a**) Regional satellite image of the study area (extracted from The Blue Marble Next Generation, NASA’s Earth Observatory^11^) (**b**) Reconstructed lake level curve of late Pleistocene Lake Lisan (upper panel)^12^, and age depth model for the Inernational Continental Scientific Drilling Program Dead Sea Deep Drilling Project (ICDP-DSDDP; lower panel)^13^. (**c**), Schematic map with ICDP-DSDDP coring location, modern mean annual rainfall, perennial streams, and the maximum extent of Lake Lisan (at 25 ka). (**d**) Satellite image of the Dead Sea and its major tributaries (created using ArcGIS® software by Esri, and includes World Imagery basemap^14^. Sources: Esri, DigitalGlobe, GeoEye, Earthstar Geographics, CNES/Airbus DS, USDA, USGS, AeroGRID, IGN, and the GIS User Community). Also depicted are the margins of Lake Lisan during the studied intervals at lake levels of 190 and 280 (m below mean sea level), depicted by blue and red lines respectively. Note the large regression of the northern shoreline during the studied time interval, whereas the eastern and western shorelines only slightly differ from the modern Dead Sea (lake level 430 mbmsl).

Hydroclimatic variability bears pronounced implications on climate characterization, and its quantification is a crucial step towards corroborating model predictions and interpreting past conditions. In arid regions, floods comprise a dominant hydroclimatic phenomenon, and often affect water availability and water management policies. Understanding how flood frequency changes with mean climatology is therefore an important, yet rarely achieved goal. In the eastern Mediterranean, reconstructing past mean centennial-millennial scale climate has become feasible through a variety of paleoclimate proxies^1–6^ (Fig. 1a). However, pinpointing flood occurrence and quantifying short-term hydroclimatic variability from geological records remains difficult, and is rarely achieved through the analyses of high-resolution archives^7–9^. Furthermore, associating synoptic-scale atmospheric circulation patterns with paleofloods is only possible where flood generation is strongly correlated with specific synoptic atmospheric patterns^10^. Determining flood frequencies during changing climate and examining if the relationship between mean climate and flood frequency holds for past climates is a crucial task for properly describing climate and preparing for the possible outcomes of climate change on *in situ* hydrology.

**Figure 2:**
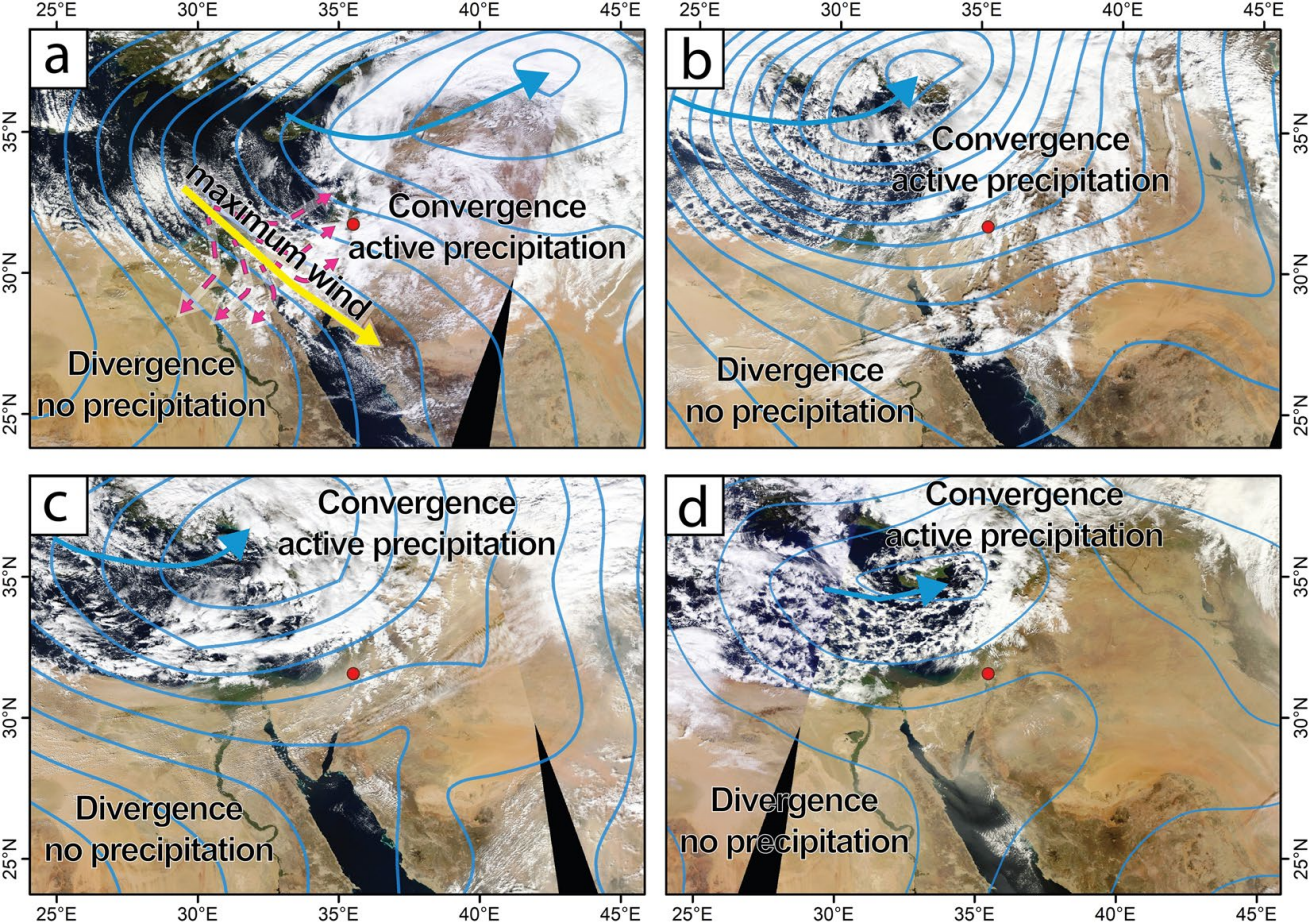
Key types of eastern Mediterranean cyclones passing through the EM, (2 hPa isobars mean sea level pressure), and past two-days cyclone trajectory (blue arrow). The cyclones form two regions of near-surface converging and diverging wind patterns, separated by a belt of maximum wind speed (yellow arrow) and dominant surface wind directions (pink dashed arrows). Precipitation is limited to convergence zones, where rapidly ascending moist air cools to form precipitating clouds. Red dot indicates location of the Inernational Continental Scientific Drilling Program Dead Sea Deep Drilling Project (ICDP-DSDDP) deep coring site. Precipitation induced by (**a**) southern-track cyclones (20-Feb 2015) and (**b**) deep cyclones (11-Dec 2010) affect the entire Dead Sea watershed, whereas (**c**) northern track-cyclones (20-Dec 2012) and (**d**) shallow cyclones (12-Feb 2015) only affect the northwestern EM, with negligible impact on the Dead Sea watershed and its tributaries. Satellite images retrieved from MODIS (Aqua/Terra) using NASA Worldview (https://worldview.earthdata.nasa.gov/). Synoptic reanalysis provided by Physical Sciences Division, Earth System Research Laboratory, NOAA, Boulder, Colorado (http://www.esrl.noaa.gov/psd/).

In the eastern Mediterranean, mean hydrological changes in the Dead Sea watershed are recorded by changing lake levels, reflecting decadal to centennial hydroclimatic trends^1,15^ (Fig. 1b,c). Dead Sea level rises (falls) were shown to reflect increased (decreased) annual precipitation, and are strongly correlated with increased (decreased) frequency of mid-latitude eastern Mediterranean cyclones (EM cyclones)^15–17^. The sedimentary record of the Dead Sea therefore provides an opportunity to test whether changes in mean precipitation are also associated with changes in flood frequency. In this paper, we present evidence corroborating the notion that flood occurrence in the EM is strongly correlated with mean climatology and that flood occurrences are non-stationary and comprise intervals of intense flooding due to changes in the track, frequency and depth of mid-latitude EM cyclones^18–20^ (Fig. 2).

## Flood climatology, lake levels and the geological record of the Dead Sea

The mean hydrological budget of the Dead Sea and its level are controlled by EM cyclones delivering ca. 90% of annual precipitation to the northern watershed, currently reaching the lake through its main water source, the perennial Jordan River^15,17,19,21,22^. EM cyclones also induce rainstorms that affect tributaries draining directly into the Dead Sea, capable of producing floods^23^ (Fig. 1d). Under modern conditions, 15–20 EM cyclones affect the northern wetter parts of the eastern Mediterranean during 45–60 days of the rainy season (October-May)^21,24,25^, whereas the arid southern and eastern parts of the watershed experience up to seven precipitating storms on average, each lasting up to two days^26^. Other synoptic-scale systems capable of generating floods are Active Red Sea troughs (ARST) and Tropical Plumes (also named Subtropical Jet-related disturbances)^20,27^. Active Red Sea troughs are associated with short-duration heavy-precipitation storms^10,28–31^, that mostly generate low-volume, scattered local floods^31,32^, whereas tropical plumes are rare, and generate widespread floods throughout the region^33–36^. The relationship between Dead Sea levels and modern regional winter rainfall, dictated by eastern Mediterranean climatology has been shown to stretch into the Holocene^15,16^, thus suggesting that elevated lake levels during the Last Glacial Maximum (Fig. 1b) also result from slight modulations of winter climatology, namely increased frequency of deep EM cyclones^16,37^ with a southward displacement of their storm track (Fig. 2)^38–40^.

The sedimentary record of the Dead Sea and its Quaternary predecessors^41^ recovered from the deepest part of the lake by the Inernational Continental Scientific Drilling Program Dead Sea Deep Drilling Project (ICDP-DSDDP) (Figs 1, S1–2), archive the regional hydroclimatic conditions of the eastern Mediterranean in annual to millennial scales^42,43^. The sedimentary deposits of Lake Lisan, the Pleistocene predecessor of the Dead Sea (ca. 85–14 ka^12,44^), ranges in thickness from 20–40 m in its margins^12,45–48^, to 110 m in the deep basin^42,44^. Varved segments^45,46,49–52^ are comprised of alternating authigenic aragonite, deposited during the dry season^53–56^, and fine-grained allochthonous detritus, delivered into the lake by floods during the rainy season^42,51,57–60^.

## Results

**Figure 3:**
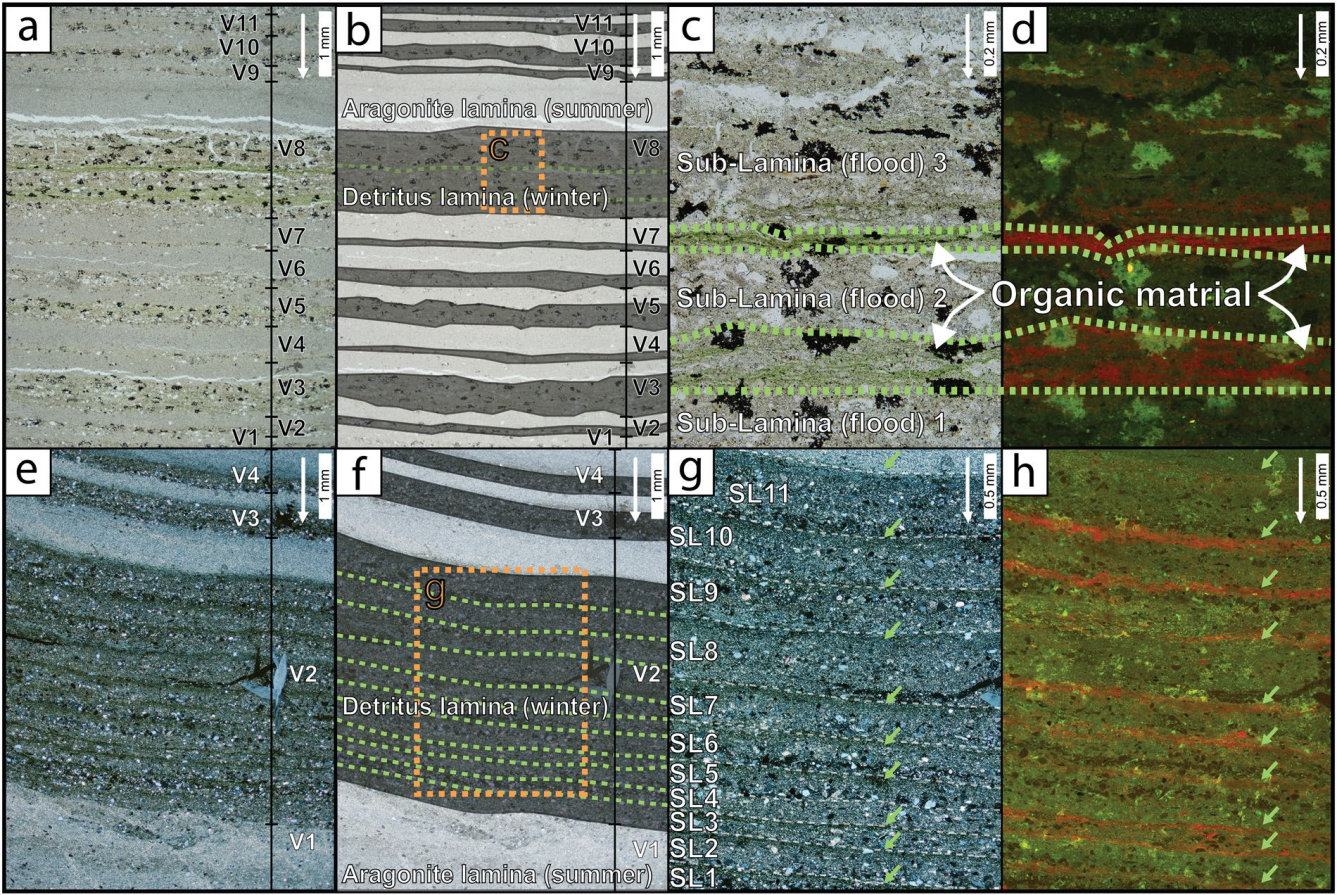
Photomicrographs taken using plane polarized, crossed polarized and fluorescence microscopy. Scale is located in the upper right corner, and white arrows next to it point in down core direction. Varves (V#), laminae and sub-laminae (SL#) are indicated in (**a, b**, **c, e, f** and **g**). (**a**), Plane polarized photomicrograph from core section 48-2 (during lake level drop) of alternating aragonite and detritus. (**b**) Same as **a** with interpretation based on microfacies analyses, one varve comprises a detrital lamina topped by an aragonite lamina, and corresponds to one year of deposition; dashed green lines indicate organic-enriched laminae. (**c**) Enlarged section of a multi-laminae detrital layer (orange dashed rectangle in b), showing thin green organic-enriched laminae between detrital sub-laminae. (**d**) Same as **c**, but under fluorescent microscopy, showing red-fluorescent organics possibly containing chlorophyll. (**e**) Plane polarized photomicrograph from core section 51-1 (during lake level rise) of alternating aragonite and detritus. (**f**) Same as e, with interpretation based on microfacies analyses, one varve corresponds to one year of deposition; dashed green lines indicate organic-enriched laminae. (**g**) Enlarged section of a multi-laminae detrital layer (orange dashed rectangle in **f**), made of 11 fining upward sub-laminae, each capped by green thin organic-enriched lamina. (**h**) Same as in **g**, but under fluorescent microscopy, showing red-fluorescence possibly containing chlorophyll.

**Figure 4:**
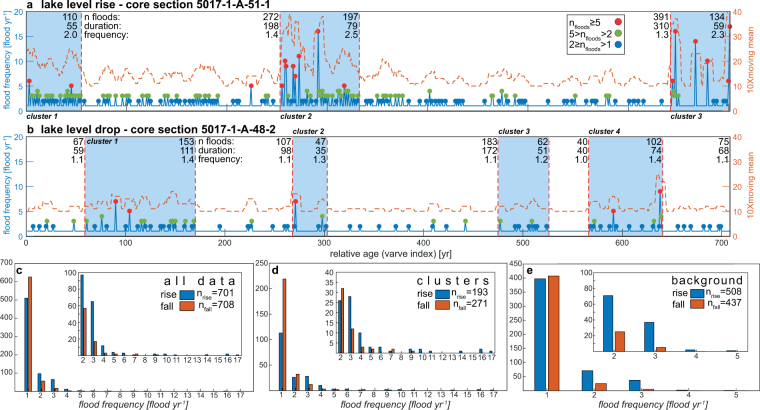
Time series and summary of annual flood frequency within varves of alternating aragonite and detritus in core sections 51-1 (**a**; lake level rise), and 48-2 (**b**; lake level drop), depicting years of frequency ≥2 (blue circles), ≥3 (green circles) and ≥5 (red circles). Orange dashed line indicates moving average of annual flood frequency multiplied by 10. Blue rectangles depict flood-rich clusters, determined by running Mann-Whitney and Ansari-Bradley tests (window width of 75 yr; Fig. S5). Total number of recorded floods, duration (in years) of cluster and background intervals, as well as mean flood frequency (floods yr^−1^) for every interval, are provided in the upper part of the plot. Summary of annual flood frequency distribution for all data (**c**), cluster intervals (**d**), and background intervals (**e**) (i.e. non-cluster periods). Respective insets in **c,** **d** and **e** depict same data, but without years of single floods. Note the significant difference between level drop and rise, indicated by the number of year of ≥2 floods and their non-uniform spread through the record.

In this study, micro-facies analysis of two sections of the DSDDP core (ca. 27 and 18 ka) reveal for the first time that these clastic laminae are comprised of several graded sub-laminae, indicating discrete and countable floods within a single wet season (Fig. 3). Significant differences in flood frequency characterize the opposing climates, reflected in lake level rises and falls during the late Pleistocene. Episodes of rising lake levels are characterized by higher frequency of floods, shorter intervals between years of multiple floods, and a significantly larger number of years that experienced multiple floods (Fig. 4; Tables S1, S2). The two studied intervals (core 5017-1-A, sections 48-2 and 51-1, 18 and 27 ka, respectively; Fig. S2) and their respective flood time series demonstrate pronounced non-stationarity, dividing the studied intervals into flood-rich clusters and background intervals (Figs 4, S3). Clusters are characterized by pronounced increased flood frequency compared with their respective background intervals, with significantly higher flood frequencies during rising lake level (Table S2). Several key differences are noted: (a) significantly more detrital laminae with ≥2 floods per year during lake level rises (27% vs. 12%; p-value < 0.01; Table S1), representing a 30% surplus in recorded floods during lake level rise (1104 vs. 836; Table S1). (b) Years with ≥2 floods per year cluster into intervals of similar mean duration (*ca*. 65 yr) during both lake level falls and rises. (c) Flood clusters are marked by +100% and +40% increased flood frequency relative to background levels, during level rise and level fall, respectively. (d) The average time interval ($\bar{\Delta }$) between consecutive years characterized by ≥2, ≥ 3, and ≥5 floods per year are 1.5-2 times longer during background periods compared with flood clusters, and more importantly, they are 2–4 times longer during lake level fall than during lake level rise (Figs 4, S3; Table S2).

## Discussion

In this study, microfacies analyses of Dead Sea varves disentangle the complex interplay between global climate change, synoptic circulation patterns and past flood hydrometeorological variability. Intra-seasonal detrital sub-laminae reveal individual floods, deposited within a single wet season during the late Pleistocene for the first time (Fig. 3). Consideration of the strong seasonal Mediterranean climate, in which rainfall is limited to the wet-season^17,61^, point to the following hydroclimatic-lacustrine sedimentary sequence:

1. Intense rainstorms in large tributaries east and west of the Dead Sea produced floods of sufficient intensity and volume, capable of carrying suspended load into the ICDP-DSDDP coring location at the lake center (Figs 1d, S1)^20,27,62^. Establishing the threshold of these floods remains beyond the scope of this paper, but it is suggested that they were larger than common gauged floods in the modern record (Fig. S4).
2. Coarse bedload was deposited in the sub-lacustrine canyons and tributary mouths, forming coarse-clastic fan-deltas and shore deposits^63,64^, whereas the silt-dominated suspended load was carried as sediment plumes towards the center of the lake, where it was deposited as thin (ca. 20–500 μm) graded sub-laminae^55,57,65^ (Figs 3; S5–6).
3. Most sub-laminae are capped by organic-rich laminae of few μm thickness, resulting from the deposition of suspended terrestrial plant remains, coupled with proliferation of epilimnion algae in response to incoming nutrient-rich floodwaters, diluting the saline lake waters, as reported in rare modern observations^66–68^ (Fig. 3).
4. Incoming floodwaters replenished the lake’s epilimnion with bi-carbonate and other salts, supporting the lake’s primary productivity and enabling aragonite precipitation during the subsequent dry season^53,54,69,70^.

Among the potential flood-producing synoptic circulation patterns, EM cyclones are the most likely candidates for generating high-sediment yield floods, and are limited between October and May^23,27,62^ (Fig. 2). During the Last Glacial Maximum (LGM), lake-level reached a maximum of 160 mbmsl^1,12^ (*ca*. 250 above modern level), and the northern and southern shores of Lake Lisan were *ca*. 150 and 90 km away from the coring site, respectively (Figs 1, S2). Furthermore, the epilimnion of Lake Lisan was significantly fresher (0–100 TDS)^4^ than modern Dead Sea brine (240 TDS)^71^. Thus, fine-grained flood-borne detritus could have reached the coring site only by floods from the larger tributaries entering the lake from the steep eastern and western escarpments^37^ (Figs 1d, S5–6).

Because mean hydroclimatic conditions and large floods in the eastern and western tributaries are generated by EM cyclones^15,16^, mean climatic conditions can now be associated with changes in the frequency of flood-producing EM cyclones during the LGM^23,27^. More specifically, EM cyclones have distinguished dry (divergence) and precipitating (convergence) regions, separated by a belt of maximal wind speed^18^ (Fig. 2a). Major precipitation events over the Dead Sea watershed occur when: (a) the cyclone is characterized by a southern track (i.e. the cyclone center passes through the EM towards the Syrian desert; Fig. 2a)^20^; or (b) the cyclone is deep enough so its effect extends south into the arid regions of the Negev desert (Fig. 2b)^19,21^. Shallow cyclones and cyclones characterized by a northern track, on the other hand, only affect the northwestern EM, with negligible impact on the Dead Sea watershed and its tributaries (Fig. 2c,d). Under modern conditions, these headwaters (at altitude of 600–800 m) are occasionally covered by snow, and since late Pleistocene winters were colder^72,73^, with more frequent snowfall in lower altitudes, EM cyclones were prone to generate snow and rain over snow at the southern mountainous areas, east and west of the Dead Sea, thus facilitating sediment transport into the deep lake (Figs S5–6). During wetter conditions, characterized by increased frequencies of deep and southern-track EM cyclones, frequencies of years with ≥2 floods per year in both background and cluster intervals significantly increased, meaning that more floods reached the coring site in the lake’s center within a single season (Tables S1-2). In contrast, when the frequency of EM cyclones decreased and lake levels dropped accordingly, frequency of years with ≥2 floods per year decreased as well. Regionally, the well-established association of increased flood frequency with lake level rise during the last glacial supports earlier assertions that level rises in this basin are primarily the product of increased precipitation.

The increased (decreased) frequency of recorded floods per year and their causative synoptic-scale atmospheric patterns during rising (falling) lake levels illustrate that during the LGM, flood hydrometeorology is embedded in- and coupled with mean hydrological conditions and climatology of the eastern Mediterranean. Hence, significantly more years experienced multiple floods during lake level rise (191 years out of 701) than during lake level drop (82 years out of 708; p-value < 0.01; Table S1). Finally, some of the studied wet winters present significantly larger number of floods per year (>10) relative to modern observations (Fig. 4), demonstrating the essential role of paleoflood records in complementing modern hydrometeorological records for improving our understanding of hydrometeorological variability. Clustering of wet seasons with ≥2 floods demonstrates that the effect of climate change on *in situ* hydrometeorological variability and flood occurrence is non-stationary and non-linear.

The study of weather, synoptic circulation patterns and the hydrological cycle *in situ* during global climate change is predominantly limited to modeling. Testing climate models capabilities in capturing hydroclimatic variability such as short-term flood clustering should involve verification against suitable records. Analyses of the seasonal to multi-annual record of Lake Lisan reveals the underlying processes comprising centennial scale climatology. This information can be introduced into climatic models to improve understanding and quantification of natural weather and climate variability. Because high-resolution archives of hydroclimatic variability are scarce, while reconstructed lake levels often smooth high-resolution hydroclimatic variability, this record can be further exploited to interpret and to model short-term hydroclimatic variability through the entire watershed during episodes of climate change.

## Materials and Methods

### Age-Depth model of the ICDP DSDDP Core 5017-1

Site 5017-1 of the ICDP DSDDP is located in the deepest floor of the Dead Sea (Fig. S1; 31°30′28.98″N\35°28′15.60″E). The core was extracted by the U.S. Drilling, Observation, and Sampling of the Earth’s Continental Crust (DOSECC) using the Deep Lake Drilling System on a barge from November 2010 to January 2011. The composite 5017-1 profile comprises authigenic halite, gypsum and aragonite as well as clastic material^42^ (Fig. S2). Some muddy segments of the core, primarily during MIS2-4, comprise alternating aragonite (authigenic) and detritus (alochtonous) forming annual varves^50,74^, in places disturbed by slump sediments^44^. Aragonite precipitates from lake waters during dry season evaporation, and detritus is deposited by floods during the rainy season^45,55,57^. Age-depth model for the ICDP 5017-1 core is based on 54 calibrated ^14^C ages using Markov chain Monte-Carlo simulation, adapted to provide a monotonic smooth spline curve (95.4% confidence interval)^13^.

### Thin sections of the ICDP-DSDDP cores

Two core sections (51-1 and 48-2) of the ICDP Dead Sea Deep Drilling Project (ICDP-DSDDP) core (Fig. S2), coeval with significant lake level rise and fall, as determined by radiocarbon chronology^13^, were selected for this study. Core sections selection was based on the availability of non-disturbed varved segments of sufficient length that also had good age constrains, with ^14^C ages extracted from within the studied core sections (Fig. S1). The sections represent similar time spans of approximately 700 years (708 and 701 years for lake level drop and rise, respectively) and were continuously sampled for thin sections using the standard procedure^75^ adjusted for salty sediments. In total, 33 large-scale thin-section samples (10 × 2 cm) were prepared; 18 sections representing lake level rise, and 15 lake level fall. The thin sections were scanned and examined using a petrographic polarized microscope (Leica DMLP) and fluorescence microscopy operated with violet and polarized light conditions (Nikon AZ100M). Images were taken with a digital camera (Olympus DP72) and processed with Nikon photo software (NIS Elements AR 4.3). These segments contain both laminated varves of alternating aragonite and detritus and mass transport layers, related to slope instability. We avoided the mass transport layers and focused on annually laminated segments to properly consider the annual sedimentary cycle and establish flood frequencies (Figs 3, S2).

### Varve counting and microfacies analyses

Varve counting was carried out under plane- and cross-polarized microscope. The common varve comprises a graded sequence capped by a thin lamina of organic-rich material. However, some detrital laminae are contain more than one graded sequence and show a multiple sub-laminae structure, which represents repeated individual floods. To avoid over-identification of sub-laminae, all sections were examined twice, and the counting of sub-laminae was conducted in a conservative fashion, so when the number of sub-laminae was questionable (within ±1 sub-laminae), the lower number of sub-laminae was used. Due to the varved nature of the studied laminated segments, a minimum number of one flood per wet season is assigned to each varve. Although we assume that some years probably experienced floods too small to reach the coring location, the nature of the sediments, does not allow an objective identification of years without any floods. This limitation results in inherited bias of the data were possible zero-floods laminae were assigned a minimum value of 1. We therefore adapted our statistical analyses and focused on years with ≥2 floods per year.

### Statistical analyses

In total, 708 and 701 varves were counted in the segments representing lake level drop and rise, respectively. The populations of the episodes representing rising and dropping lake levels were compared with Matlab©^76^ and R^77^ using Kolmogorov-Smirnov^78^, Mann-Whitney (Wilcoxon)^79^, Ansari-Bradley^80^, chi-squared^81^ and logistic regression^82,83^ tests to determine the significance of the difference between the two segments. The difference between the populations is significant in all of the above-mentioned tests. A negative regression coefficient of −0.51 in the logistic regression model further emphasizes that the probability for a detritus laminae to be associated with period of rising lake level increases with the number of sub-laminae. Identification of clusters was carried out in a semi-objective approach with the consideration of running Mann-Whitney and Ansari-Bradley tests with a window width of 75 yrs (Fig. S3), along with the moving average and its first and second derivatives.

### Statistical tests carried out with Matlab^©^ and R

Applied code for statistical analyses. Software environment used (R/Matlab) is noted for each analysis^76,77^.

#### Chi2 (on lumped contingency table); R

- *chisq.test*(*rise, drop, correct* = *FALSE*)

Pearson’s Chi-squared test

X-squared = 62.244, df = 4, **p-value = 9**.**786e-13**

#### Mann-Whitney (on original data); Matlab

- [*p,h*] = *ranksum*(*rise,drop*);

**p = 1**.**7390e-14**

#### Ansari-Bradley (on original data); Matlab

- [*h,p*] = *ansaribradley(rise-median(rise),drop-median(drop))*;

**p = 4**.**3361e-15**

**Pr(>F)**

–

#### Kolmogorov-Smirnov test (on original data); R

- *ks.test(drop,rise)*

Two-sample Kolmogorov-Smirnov test

data: drop and rise

D = 0.12954, **p-value = 1**.**468e-05**

alternative hypothesis: two-sided

Warning message:

In ks.test(drop, rise):

p-value will be approximate in the presence of ties

#### Logistic regression (using contingency table); R

- *anova(glm(cbind(drop,rise)~laminae, family* = *binomial), test* = *“Chisq”)*
  Analysis of Deviance Table
  Model: binomial, link: logit
  Response: cbind(drop, rise)
  Terms added sequentially (first to last)
  Df Deviance Resid. Df Resid. Dev **Pr(>Chi)**
  NULL 11 73.319
  laminae 1 53.723 10 19.596 **2**.308e-13 ***
  –
  Signif. codes: 0 ‘***’ 0.001 ‘**’ 0.01 ‘*’ 0.05 ‘.’ 0.1 ‘’ 1
- *summary(glm(cbind(drop,rise)~laminae,family* = *binomial))*

Call:

glm(formula = cbind(drop, rise) ~ laminae, family = binomial)

Deviance Residuals:

Min 1Q Median 3Q Max

−1.85743 −0.37958 −0.03628 0.78939 2.85062

Coefficients:

Estimate Std. Error z value **Pr(>|z|)**

(Intercept) 0.6795 0.1201 5.660 1.52e-08 ***

laminae **−0**.**5123** 0.0858 −5.971 **2**.36e-09 ***

–

Signif. codes: 0 ‘***’ 0.001 ‘**’ 0.01 ‘*’ 0.05 ‘.’ 0.1 ‘’ 1

(Dispersion parameter for binomial family taken to be 1)

Null deviance: 73.319 on 11 degrees of freedom

Residual deviance: 19.596 on 10 degrees of freedom

AIC: 49.181

Number of Fisher Scoring iterations: 5

## Electronic supplementary material

supplementary tables and figures

## References

[1] Torfstein, A. & Enzel, Y. In Quaternary of the Levant: Environments, Climate Change, and Humans (eds Ofer Bar-Yosef & Yehouda Enzel) 107–114 (Cambridge University Press, 2017).

[2] Torfstein A (2015). Dead Sea drawdown and monsoonal impacts in the Levant during the last interglacial. Earth Planet. Sci. Lett..

[3] Litt T, Ohlwein C, Neumann FH, Hense A, Stein M (2012). Holocene climate variability in the Levant from the Dead Sea pollen record. Quaternary Science Reviews.

[4] Begin ZB (2004). Southward migration of rain tracks during the last glacial, revealed by salinity gradient in Lake Lisan (Dead Sea rift). Quaternary Science Reviews.

[5] Goldsmith Y, Polissar P, Ayalon A, Bar-Matthews M, Broecker W (2017). The modern and Last Glacial Maximum hydrological cycles of the Eastern Mediterranean and the Levant from a water isotope perspective. Earth Planet. Sci. Lett..

[6] Palchan, D. *et al*. North Atlantic controlled depositional cycles in MIS 5e layered sediments from the deep Dead Sea basin. Quaternary Research **87**, 168–179 (2017).

[7] Anderson RY, Dean WE (1988). Lacustrine varve formation through time. Palaeogeography, Palaeoclimatology, Palaeoecology.

[8] Dean, W. E., Bradbury, J. P., Anderson, R. & Barnosky, C. The variability of Holocene climate change: evidence from varved lake sediments. (1984). 10.1126/science.226.4679.1191 17770990

[9] Ojala A, Francus P, Zolitschka B, Besonen M, Lamoureux S (2012). Characteristics of sedimentary varve chronologies–a review. Quaternary Science Reviews.

[10] Ahlborn Marieke (2018). Increased frequency of torrential rainstorms during a regional late Holocene eastern Mediterranean drought. Quaternary Research.

[11] Stöckli, R., Vermote, E., Saleous, N., Simmon, R. & Herring, D. The Blue Marble Next Generation-A true color earth dataset including seasonal dynamics from MODIS. Published by the NASA Earth Observatory (2005).

[12] Torfstein A, Goldstein SL, Kagan EJ, Stein M (2013). Integrated multi-site U–Th chronology of the last glacial Lake Lisan. Geochim. Cosmochim. Acta.

[13] Kitagawa, H. *et al*. In Radiocarbon Conference 1–12 (University of Arizona, Dakar, Senegal, 2016).

[14] ESRI. World Imagery (2009).

[15] Enzel Yehouda, Bookman (Ken Tor) Revital, Sharon David, Gvirtzman Haim, Dayan Uri, Ziv Baruch, Stein Mordechai (2003). Late Holocene climates of the Near East deduced from Dead Sea level variations and modern regional winter rainfall. Quaternary Research.

[16] Enzel Yehouda (2008). The climatic and physiographic controls of the eastern Mediterranean over the late Pleistocene climates in the southern Levant and its neighboring deserts. Global and Planetary Change.

[17] Ziv B, Dayan U, Kushnir Y, Roth C, Enzel Y (2006). Regional and global atmospheric patterns governing rainfall in the southern Levant. International Journal of Climatology.

[18] Goldreich Y, Mozes H, Rosenfeld D (2004). Radar analysis of cloud systems and their rainfall yield in Israel. Isr. J. Earth Sci.

[19] Goldreich, Y. Spatial Distribution of Mid-season Rainfall Date in Israel - a Review. Horizons in Geography, 177–182 (2004).

[20] Kahana R, Ziv B, Enzel Y, Dayan U (2002). Synoptic climatology of major floods in the Negev Desert, Israel. International Journal of Climatology.

[21] Saaroni H, Halfon N, Ziv B, Alpert P, Kutiel H (2010). Links between the rainfall regime in Israel and location and intensity of Cyprus lows. International Journal of Climatology.

[22] Zappa G, Hawcroft MK, Shaffrey L, Black E, Brayshaw DJ (2015). Extratropical cyclones and the projected decline of winter Mediterranean precipitation in the CMIP5 models. Climate Dynamics.

[23] Belachsen, I., Marra, F., Peleg, N. & Morin, E. Convective rainfall in dry climate: relations with synoptic systems and flash-flood generation in the Dead Sea region. Hydrol. Earth Syst. Sci. Discuss (2017).

[24] Alpert P, Osetinsky I, Ziv B, Shafir H (2004). Semi‐objective classification for daily synoptic systems: Application to the eastern Mediterranean climate change. International Journal of Climatology.

[25] Alpert P, Osetinsky I, Ziv B, Shafir H (2004). A new seasons definition based on classified daily synoptic systems: an example for the eastern Mediterranean. International Journal of Climatology.

[26] Aviad Y, Kutiel H, Lavee H (2013). Empirical models of rain-spells characteristics–A case study of a Mediterranean-arid climatic transect. Journal of arid environments.

[27] Dayan U, Morin E (2006). Flash flood–producing rainstorms over the Dead Sea: A review. Geological Society of America Special.

[28] Ashbel D (1938). Great floods in Sinai Peninsula, Palestine, Syria and the Syrian desert, and the influence of the Red Sea on their formation. Quarterly Journal of the Royal Meteorological Society.

[29] Vries A (2013). Extreme precipitation events in the Middle East: dynamics of the Active Red Sea Trough. Journal of Geophysical Research: Atmospheres.

[30] Krichak S, Alpert P, Krishnamurti T (1997). Interaction of topography and tropospheric flow—a possible generator for the Red Sea trough?. Meteorology and Atmospheric Physics.

[31] Armon, M., Dente, E., Smith, J. A., Enzel, Y. & Morin, E. Synoptic-scale control over modern rainfall and flood patterns in the Levant drylands with implications for past climates. Journal of Hydrometeorology (in press) 10.1175/JHM-D-18-0013.

[32] Shentsis I, Laronne JB, Alpert P (2012). Red Sea Trough flood events in the Negev, Israel (1964–2007). Hydrological sciences journal.

[33] Rubin S, Ziv B, Paldor N (2007). Tropical plumes over eastern North Africa as a source of rain in the Middle East. Monthly Weather Review.

[34] Ziv B (2001). A subtropical rainstorm associated with a tropical plume overAfrica and the Middle-East. Theoretical and Applied Climatology.

[35] Tubi A, Dayan U (2014). Tropical Plumes over the Middle East: Climatology and synoptic conditions. Atmospheric Research.

[36] Amit Rivka, Simhai Ori, Ayalon Avner, Enzel Yehouda, Matmon Ari, Crouvi Onn, Porat Naomi, McDonald Eric (2011). Transition from arid to hyper-arid environment in the southern Levant deserts as recorded by early Pleistocene cummulic Aridisols. Quaternary Science Reviews.

[37] Greenbaum N, Ben-Zvi A, Haviv I, Enzel Y (2006). The hydrology and paleohydrology of the Dead Sea tributaries. Geological Society of America Special Papers.

[38] Laîné A (2009). Northern hemisphere storm tracks during the last glacial maximum in the PMIP2 oceanatmosphere coupled models: energetic study, seasonal cycle, precipitation. Climate Dynamics.

[39] Ludwig P, Schaffernicht EJ, Shao Y, Pinto JG (2016). Regional atmospheric circulation over Europe during the Last Glacial Maximum and its links to precipitation. Journal of Geophysical Research: Atmospheres.

[40] Merz N, Raible CC, Woollings T (2015). North Atlantic eddy-driven jet in interglacial and glacial winter climates. Journal of Climate.

[41] Stein, M. The evolution of Neogene-Quaternary water-bodies in the Dead Sea rift valley, in Dead Sea Transform Fault System: Reviews 279-316 (Springer, 2014).

[42] Ina Neugebauer (2014). Lithology of the long sediment record recovered by the ICDP Dead Sea Deep Drilling Project (DSDDP). Quaternary Science Reviews.

[43] Stein, M. The ICDP Dead Sea Deep Drilling Project. (Geological Survey of Israel, 2012).

[44] Kagan E, Stein M, Marco S (2018). Integrated Paleoseismic Chronology of the Last Glacial Lake Lisan: From Lake Margin Seismites to Deep‐Lake Mass Transport Deposits. Journal of Geophysical Research: Solid Earth.

[45] Begin, Z., Ehrlich, A. & Nathan, Y. Lake Lisan: the pleistocene precursor of the Dead Sea. (Ministry of Commerce and Industry, Geological Survey, 1974).

[46] Begin Z, Nathan Y, Ehrlich A (1980). Stratigraphy and facies distribution in the Lisan Formation—new evidence from the area south of the Dead Sea, Israel. Israel Journal of Earth Sciences.

[47] Marco S, Stein M, Agnon A, Ron H (1996). Long‐term earthquake clustering: A 50,000‐year paleoseismic record in the Dead Sea Graben. Journal of Geophysical Research: Solid Earth.

[48] Haase-Schramm A, Goldstein SL, Stein M (2004). U-Th dating of Lake Lisan (late Pleistocene dead sea) aragonite and implications for glacial east Mediterranean climate change. Geochim. Cosmochim. Acta.

[49] Migowski C, Agnon A, Bookman R, Negendank JF, Stein M (2004). Recurrence pattern of Holocene earthquakes along the Dead Sea transform revealed by varve-counting and radiocarbon dating of lacustrine sediments. Earth Planet. Sci. Lett..

[50] Prasad Sushma (2004). Evidence from Lake Lisan of solar influence on decadal- to centennial-scale climate variability during marine oxygen isotope stage 2. Geology.

[51] Neugebauer, I. *et al*. Hydroclimatic variability in the Levant during the early last glacial (∼117–75 ka) derived from micro-facies analyses of deep Dead Sea sediments. *Climate of the Past* **12**, 75–90 (2016).

[52] Neugebauer Ina (2015). Evidences for centennial dry periods at ~3300 and ~2800 cal. yr BP from micro-facies analyses of the Dead Sea sediments. The Holocene.

[53] Friedman GM (1965). On the origin of aragonite in the Dead Sea. Israel. J. Earth Sci.

[54] Stein M (1997). Strontium isotopic, chemical, and sedimentological evidence for the evolution of Lake Lisan and the Dead Sea. Geochim. Cosmochim. Acta.

[55] Neev, D. & Emery, K. O. The Dead Sea. (1967).

[56] Neev D (1963). Recent precipitation of calcium salts in the Dead Sea. Bull. Res. Council Israel. Sect. G.

[57] Garber RA, Levy Y, Friedman GM (1987). The sedimentology of the Dead Sea. Carbonates and Evaporites.

[58] Haliva-Cohen A, Stein M, Goldstein SL, Sandler A, Starinsky A (2012). Sources and transport routes of fine detritus material to the Late Quaternary Dead Sea basin. Quaternary Science Reviews.

[59] Levy, Y. Recent depositional environments in the Dead Sea. Geological Survey of Israel (1988).

[60] Levy, I. Quality and quantity of sediments collected in sediment traps in the Dead Sea 1980/1981. (Geological Survey of Israel, 1982).

[61] Kushnir, Y., Dayan, U., Ziv, B., Morin, E. & Enzel, Y. in The Levant (eds Y Enzel & Bar-Yosef O) Ch. 4, (Cambridge University Press, 2017).

[62] Morin E, Harats N, Jacoby Y, Arbel S, Getker M, Arazi A, Grodek T, Ziv B, Dayan U (2007). Studying the extremes: hydrometeorological investigation of a flood-causing rainstorm over Israel. Advances in Geosciences.

[63] Machlus M, Enzel Y, Goldstein SL, Marco S, Stein M (2000). Reconstructing low levels of Lake Lisan by correlating fan-delta and lacustrine deposits. Quaternary International.

[64] Bartov Y, Stein M, Enzel Y, Agnon A, Reches Ze (2002). Lake levels and sequence stratigraphy of Lake Lisan, the late Pleistocene precursor of the Dead Sea. Quaternary Research.

[65] Garber, R. A. The sedimentology of the Dead Sea. (1980).

[66] Nissenbaum A, Baedecker M, Kaplan I (1972). Organic geochemistry of Dead Sea sediments. Geochim. Cosmochim. Acta.

[67] Oren A, Gurevich P, Anati DA, Barkan E, Luz B (1995). A bloom of Dunaliella parva in the Dead Sea in 1992: biological and biogeochemical aspects. Hydrobiologia.

[68] Oren, A. & Shilo, M. Population dynamics of Dunaliella parva in the Dead Seal. LIMNOLOGY 27 (1982).

[69] Luz, B., Stiller, M. & Talma, S. Carbon dynamics in the Dead Sea In: Niemi TM, Ben-Avraham Z., Gat J.R., editors. The Dead Sea: The Lake and Its Setting, 171–183 (1997).

[70] Barkan E, Luz B, Lazar B (2001). Dynamics of the carbon dioxide system in the Dead Sea. Geochim. Cosmochim. Acta.

[71] Sirota I, Arnon A, Lensky NG (2016). Seasonal variations of halite saturation in the Dead Sea. Water Resources Research.

[72] Affek HP, Bar-Matthews M, Ayalon A, Matthews A, Eiler JM (2008). Glacial/interglacial temperature variations in Soreq cave speleothems as recorded by ‘clumped isotope’thermometry. Geochim. Cosmochim. Acta.

[73] Bar-Matthews, M., Ayalon, A., Vaks, A. & Frumkin, A. In Quaternary of the Levant: Environments, Climate Change, and Humans (eds Ofer Bar-Yosef & Yehouda Enzel) 145-150 (Cambridge University Press, 2017).

[74] Machlus, M. Geochemical parameters in the Lisan Formation aragonite—Proxies for paleolimnology of Lake Lisan and climatic history of the Dead Sea region [M. Sc. Thesis]: The Hebrew University of Jerusalem, 81 p M. Sc. thesis, (1996).

[75] Brauer A, Endres C, Negendank JF (1999). Lateglacial calendar year chronology based on annually laminated sediments from Lake Meerfelder Maar, Germany. Quaternary International.

[76] MATLAB and Statistics Toolbox (The MathWorks inc., Natick, Massachusetts, United States, 2016).

[77] R: A language and environment for statistical computing (R Foundation for Statistical Computing, Vienna, Austria, 2016).

[78] Birnbaum Z, Tingey FH (1951). One-sided confidence contours for probability distribution functions. The Annals of Mathematical Statistics.

[79] Mann, H. B. & Whitney, D. R. On a test of whether one of two random variables is stochastically larger than the other. The annals of mathematical statistics, 50–60 (1947).

[80] Ansari AR, Bradley RA (1960). Rank-sum tests for dispersions. The Annals of Mathematical Statistics.

[81] Agresti, A. An introduction to categorical data analysis, 2nd edn. Hoboken. (NJ: Wiley-Interscience, 2007).

[82] Venables, W. N. & Ripley, B. D. Modern applied statistics with S-PLUS. (Springer Science & Business Media, 2013).

[83] McCullagh, P. & Nelder, J. A. Generalized Linear Models, no. 37 in Monograph on Statistics and Applied Probability. (1989).

