# Supplementary material for: Changing flood frequencies under opposing late Pleistocene eastern Mediterranean climates
Source: Sci Rep. 2018 May 31;8:8445. doi: 10.1038/s41598-018-25969-6 (PMC5981606; doi:10.1038/s41598-018-25969-6)
Supplement: Supplementary file 1 — supplementary tables and figures [file 41598_2018_25969_MOESM1_ESM.docx]

Drastic changes in flood frequencies under drier vs wetter late Pleistocene eastern Mediterranean climates

## Authors

Yoav Ben Dor*^1^, Moshe Armon^1^, Marieke Ahlborn^2^, Efrat Morin^1^, Yigal Erel^1^, Achim Brauer^2^, Markus J. Schwab^2^, Rik Tjallingii^2^, Yehouda Enzel^1^

## Supplementary Material

### Supplementary Tables

#### Table S1 – microfacies analysis of detrital layers indicating number of sub-laminae in clastic varves. For the X^2^ test, categories with ≥5 floods were merged (X^2^ = 62.244, DF = 4, p-value = 9.786E-13).

|  | Core section 48-2  (lake level drop) | | Core section 51-1  (lake level rise) | |  |
| --- | --- | --- | --- | --- | --- |
| Number of sub-laminae per year/varve | **n varves** | **Total number of sub-laminae** | **n varves** | **Total number of sub-laminae** | **Excess floods**  **Rise vs. drop** |
| 1 | 626 | 626 | 510 | 510 | -116 |
| 2 | 57 | 114 | 97 | 194 | 80 |
| 3 | 17 | 51 | 65 | 195 | 144 |
| 4 | 3 | 12 | 12 | 48 | 36 |
| 5 | 2 | 10 | 4 | 20 | 10 |
| 6 | 0 | 0 | 3 | 18 | 18 |
| 7 | 2 | 14 | 1 | 7 | -7 |
| 8 | 0 | 0 | 0 | 0 | 0 |
| 9 | 1 | 9 | 2 | 18 | 9 |
| 10 | 0 | 0 | 2 | 20 | 20 |
| 11 | 0 | 0 | 1 | 11 | 11 |
| 12 | 0 | 0 | 0 | 0 | 0 |
| 13 | 0 | 0 | 0 | 0 | 0 |
| 14 | 0 | 0 | 1 | 14 | 14 |
| 15 | 0 | 0 | 0 | 0 | 0 |
| 16 | 0 | 0 | 2 | 32 | 32 |
| 17 | 0 | 0 | 1 | 17 | 17 |
| Total: | **Years (varves)** | **Sub-laminae** | **Years (varves)** | **Sub-laminae** | **Excess floods** |
|  | 708 | 836 | 701 | 1104 | 268 |

#### Table S2 – descriptive statistical parameters of duration, frequency [floods yr-1] and time interval [yr] between years with ≥2, ≥3 and ≥5 recorded floods for the studied sections of lake level rise (51-1) and lake level drop (48-2) for background and cluster periods. Note: the average time interval ($\bar{\Delta}$) between years characterized by ≥2, ≥3, and ≥5 floods are 1.5-2 times longer in background levels with respect to clusters, and more importantly, they are 2-4 times longer during falling lake level (Fig. S3). Furthermore, each of the studied intervals demonstrate a non-uniform flood distribution that does not fit a Poisson distribution (p<0.01). Cluster and background intervals individually demonstrate pronounced non-stationarity and neither fit a Poisson distribution (p<0.01).

| Core segment | lake level change |  | parameter | unit | average | median | min | max |
| --- | --- | --- | --- | --- | --- | --- | --- | --- |
| 51-1 | rise | background | duration | yr | 254.0 | 254 | 198 | 310 |
| 51-1 | rise | background | frequency | Flood yr^-1^ | 1.3 | 1.3 | 1.3 | 1.4 |
| 51-1 | rise | background | Δ (≥2 floods/yr) | yr | 4.4 | 4.4 | 3.8 | 5.0 |
| 51-1 | rise | background | Δ (≥3 floods/yr) | yr | 11.2 | 11.2 | 8.5 | 13.9 |
| 51-1 | rise | background | Δ (≥5 floods/yr) | yr | 179.0 | 179.0 | 179.0 | 179.0 |
| 51-1 | rise | cluster | duration | yr | 64 | 59 | 55 | 79 |
| 51-1 | rise | cluster | frequency | Flood yr^-1^ | 2.3 | 2.3 | 2.0 | 2.5 |
| 51-1 | rise | cluster | Δ (≥2 floods/yr) | yr | 3.1 | 2.3 | 1.8 | 5.2 |
| 51-1 | rise | cluster | Δ (≥3 floods/yr) | yr | 7.1 | 3.5 | 2.8 | 14.9 |
| 51-1 | rise | cluster | Δ (≥5 floods/yr) | yr | 43.4 | 42.0 | 11.6 | 76.6 |
| 48-2 | drop | background | duration | yr | 89.4 | 68 | 50 | 172 |
| 48-2 | drop | background | frequency | Flood yr^-1^ | 1.1 | 1.1 | 1.0 | 1.1 |
| 48-2 | drop | background | Δ (≥2 floods/yr) | yr | 11.0 | 10.2 | 8.2 | 15.6 |
| 48-2 | drop | background | Δ (≥3 floods/yr) | yr | 52.3 | 58.0 | 27.0 | 72.0 |
| 48-2 | drop | background | Δ (≥5 floods/yr) | yr | ~ | ~ | ~ | ~ |
| 48-2 | drop | cluster | duration | yr | 65 | 58 | 35 | 111 |
| 48-2 | drop | cluster | frequency | Flood yr^-1^ | 1.3 | 1.4 | 1.2 | 1.4 |
| 48-2 | drop | cluster | Δ (≥2 floods/yr) | yr | 7.5 | 8.0 | 5.0 | 9.1 |
| 48-2 | drop | cluster | Δ (≥3 floods/yr) | yr | 26.5 | 29.8 | 11.0 | 35.5 |
| 48-2 | drop | cluster | Δ (≥5 floods/yr) | yr | 121.5 | 167.0 | 14.0 | 183.5 |

### Supplementary Figures


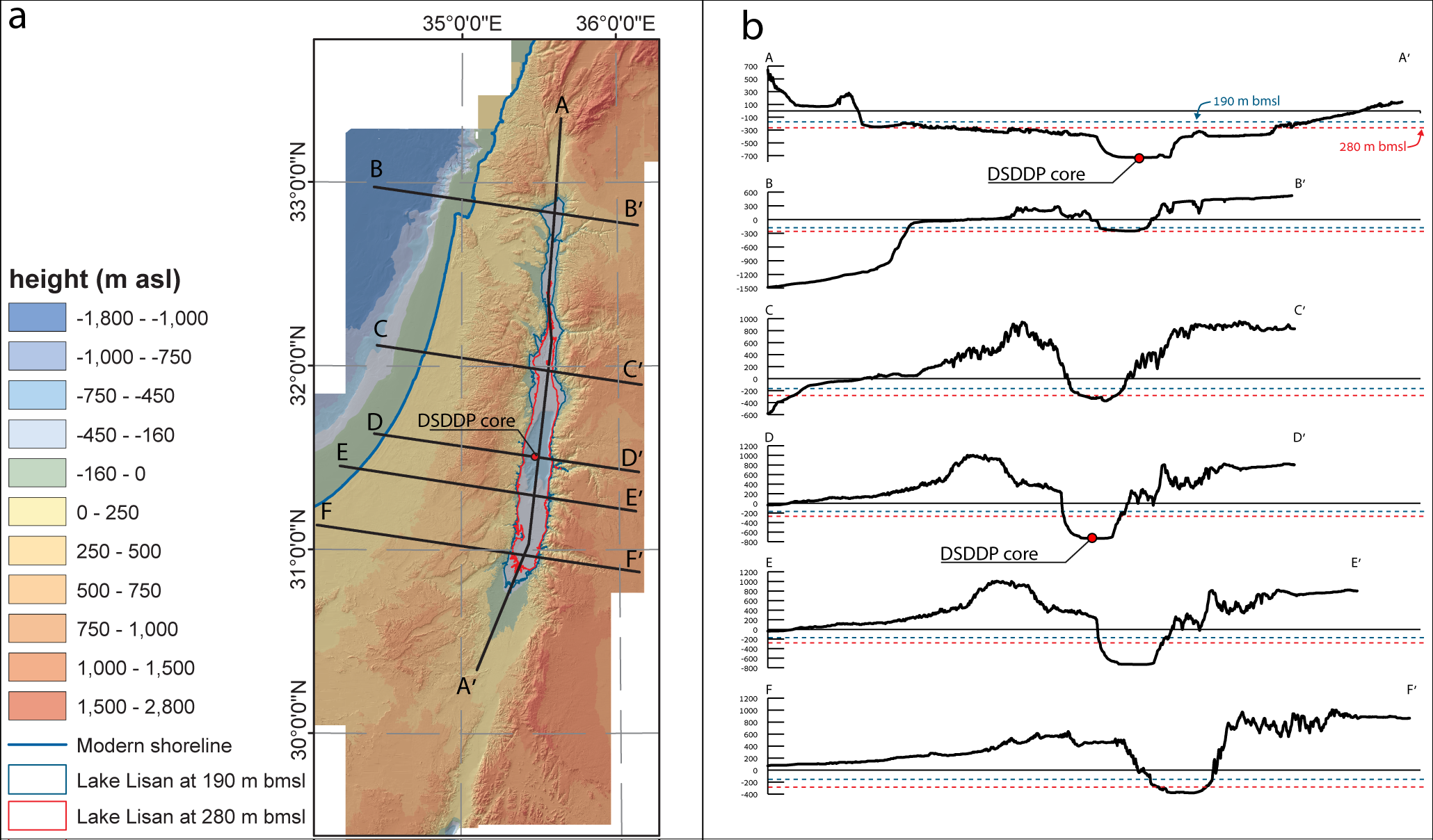


#### Figure S1 – **a,** Aerial extent of Lake Lisan during the studied intervals, with water levels at 190 (25.5 ka) and 280 (18.5 ka) mbmsl, plotted on a shaded relief map with topography^1^. **b,** representative cross-sections of the Dead Sea rift valley and its environs. Sections locations are depicted in **a**.


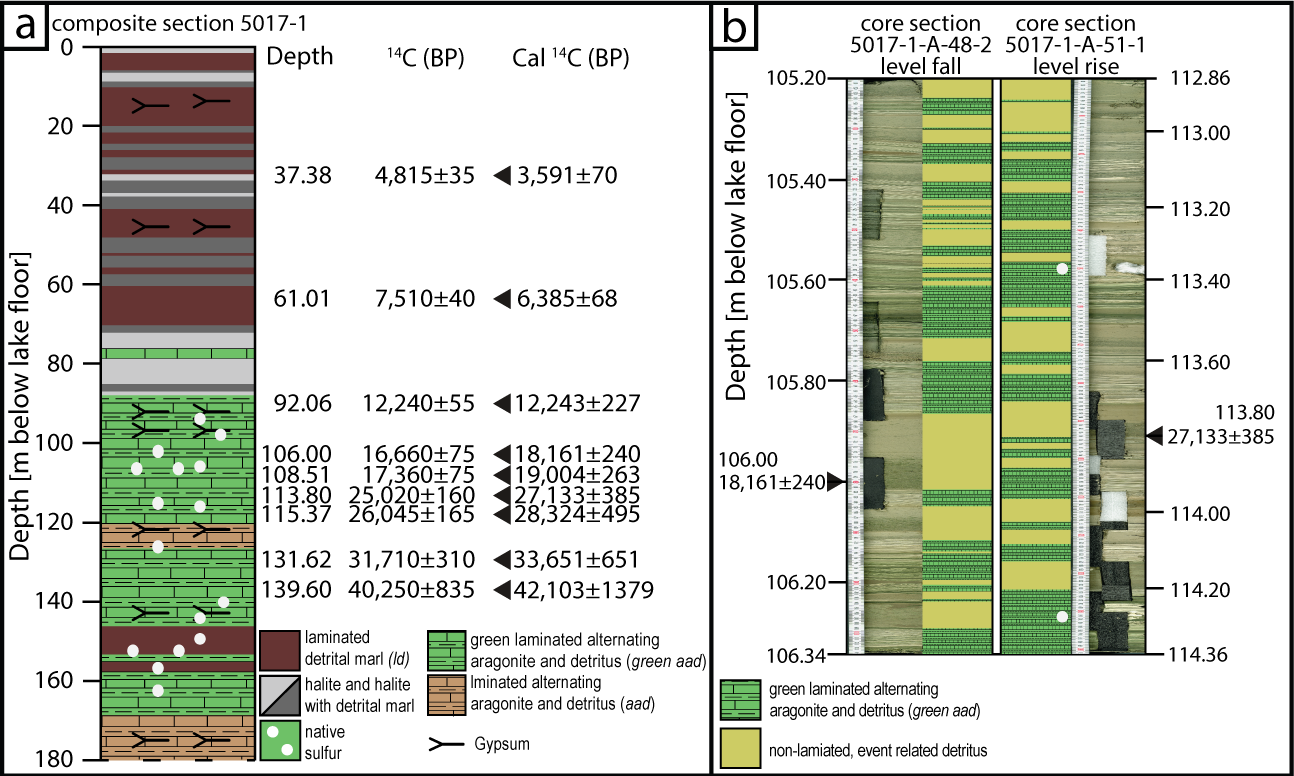


#### Figure S2 – **a,** Schematic composite lithology of the ICDP-DSDDP cores^2^ with ^14^C ages of terrestrial plant remains and corresponding depths^3^ (95.4% confidence, calibrated with IntCal13 using OxCal online^4^). **b,** Images and schematic representations of core sections 5017-A-48-2 and 5017-A-51-1, analyzed in this study, with calibrated ^14^C ages^3^ representing episodes of lake level fall and rise, respectively.


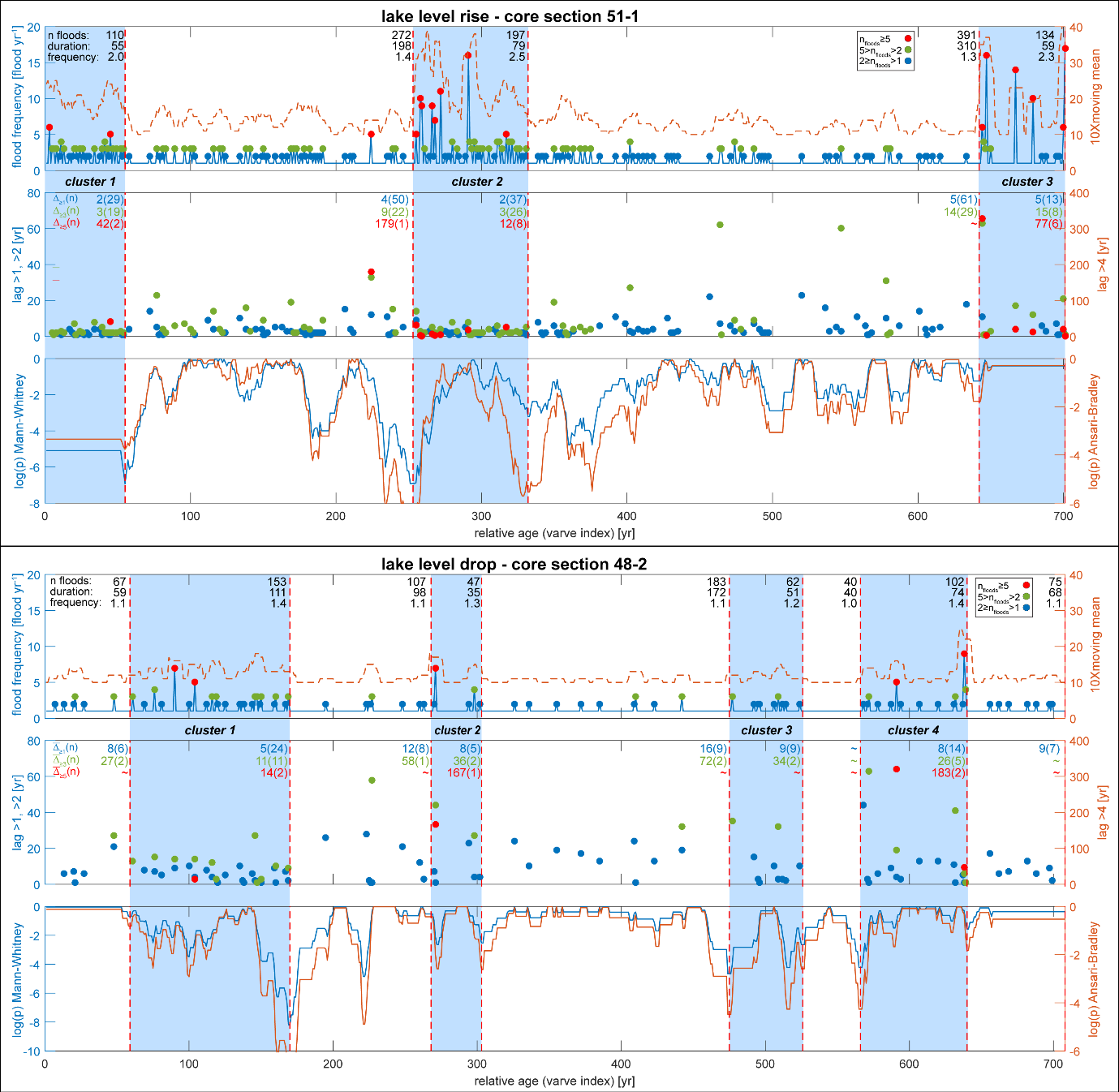


#### Figure S3 – complete time series of annual flood frequency (upper panel) within alternating aragonite and detritus facies of core segment 51-1 (lake level rise), and core segment 48-2 (lake level drop), depicting years of intensity ≥2 (blue circles), ≥3 (green circles) and ≥5 (red circles). Middle panel depicts time interval between years of intensity ≥2 (blue circles), ≥3 (green circles) and ≥5 (red circles) parenthesis indicate number of observations per segment. Blue rectangles depict clusters determined by running Mann-Whitney and Ansari-Bradley tests (lower panel; window width of 75 yr). Intense flooding clusters range between 55-79 yr (mean 64 yr) during lake level rise, and from 35-111 yr (mean 65 yr) during lake level drop, corresponding to 28% and 38% of the studied intervals. i.e., the same mean cluster duration but with a much larger variation during lake level drops.


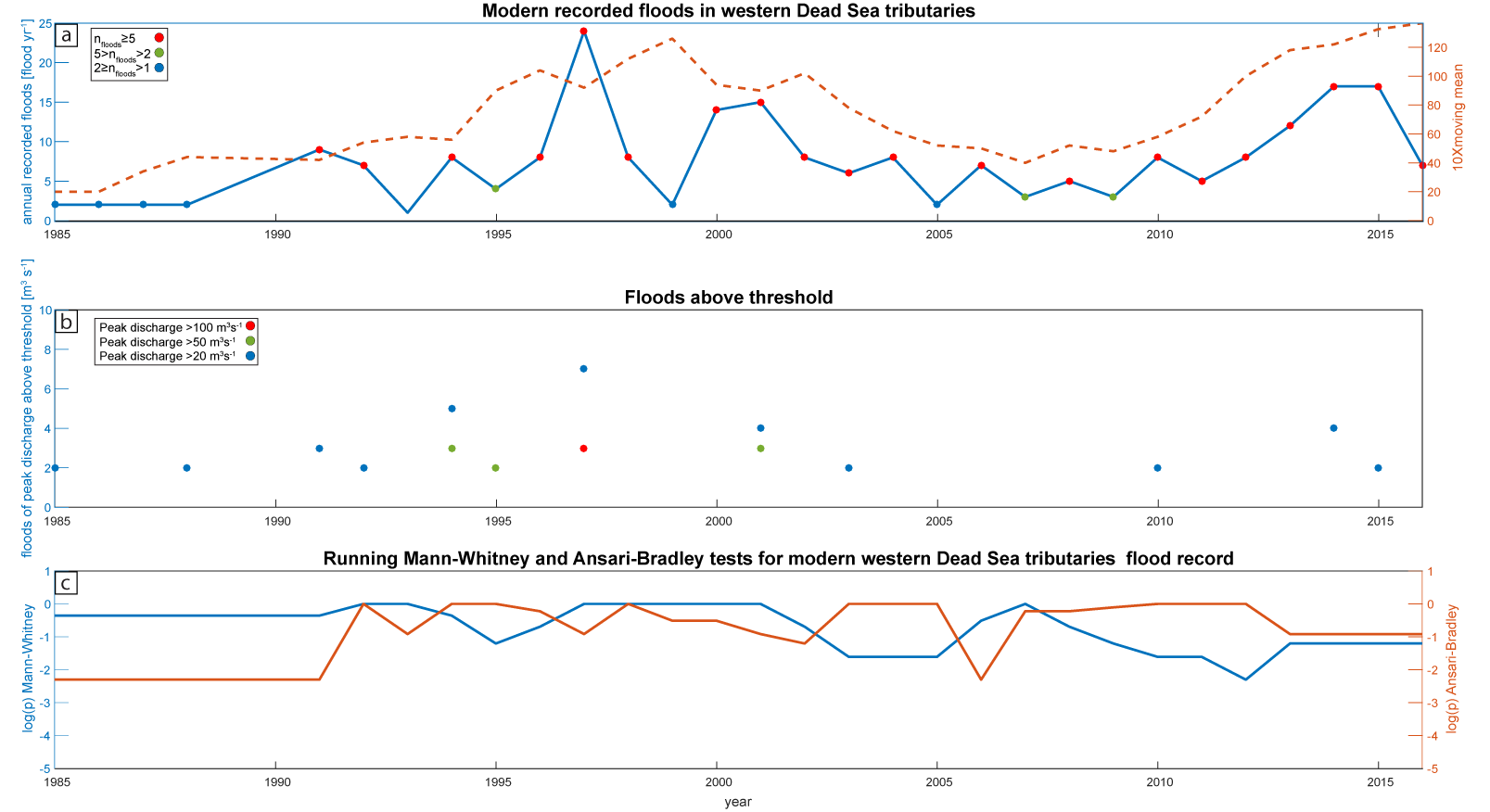


#### Figure S4 – modern record of floods in Dead Sea tributaries. **a**, time series of recorded modern annual floods in four western Dead Sea tributaries (Arugot, Teqoa, Rahaf and Darga) depicting years of flood frequency ≥2 (blue circles), ≥3 (green circles) and ≥5 (red circles). **b**, floods with peak discharge >20 [m^3^s^-1^] (blue circles), >50 [m^3^s^-1^] (green circles) and >100 [m^3^s^-1^] (red circles). **c**, running Mann-Whitney and Ansari-Bradley tests (window width 15 years) indicate no abrupt changes in time series characteristics, probably due to the limited extent of available data.

####

*
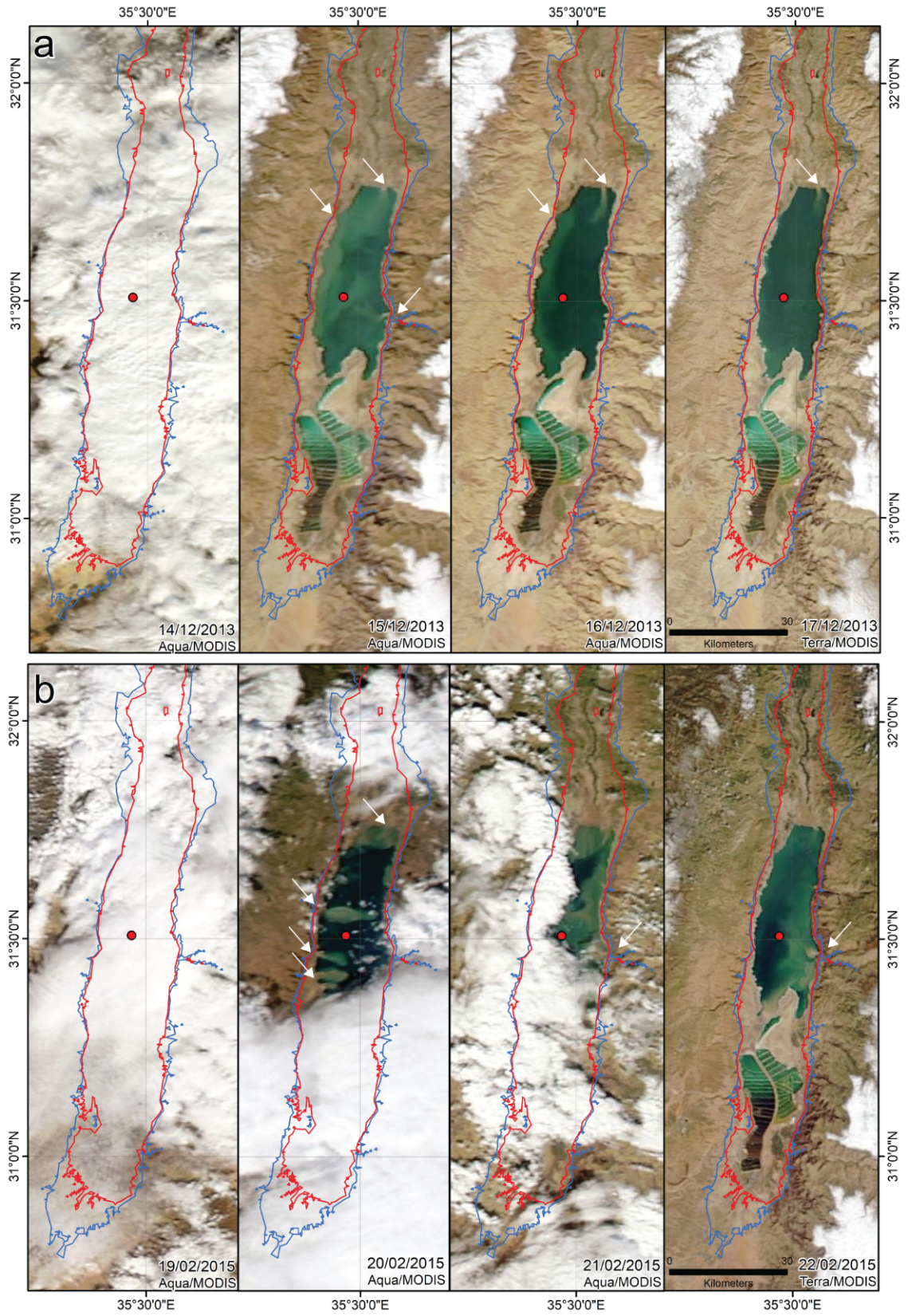
*

#### Figure S5 – flood-borne sediment plumes (indicated by arrows), some of which reach the deep Dead Sea during winters after rainfall and snow events during December 15^th^, 2013 (a) and February 21^st^, 2015 (b). Red dot indicates location of the DSDDP coring site (N 31^O^30'28.98'', E 35^O^28'15.60''). Note the extent of lake Lisan and the northward retreat of the northern shoreline for lake levels of 190 and 280 mbmsl, depicted by blue and red lines respectively. Satellite images retrieved from MODIS (Aqua/Terra) using NASA worldview (https://worldview.earthdata.nasa.gov/).


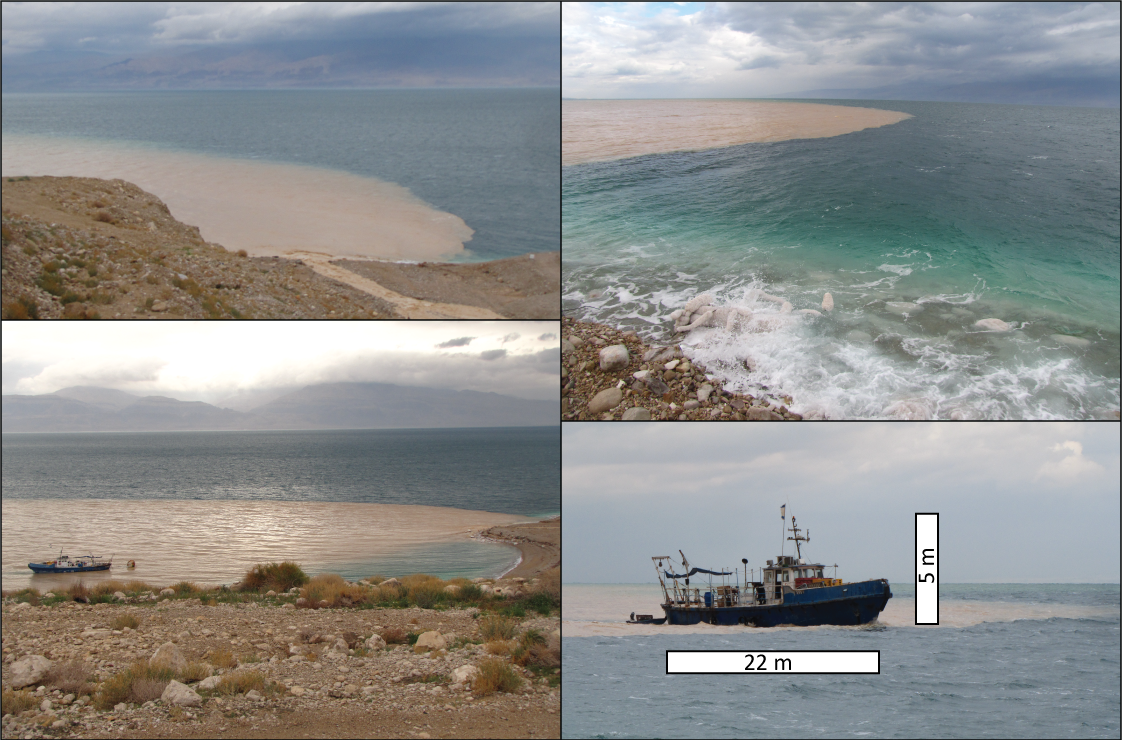


Figure S6 – sediment-rich flood waters forming a turbid sediment plume on the dense Dead Sea during 2015/2016 winter (February 22^nd^ 2016) at Nahal Arugot's mouth (near Ein Gedi, Israel; 31^O^27'36''N/35^O^23'55''E). Boat size is ca. 22 m in length and 5 m tall. Photo credit: Liran Ben Moshe and Ido Sirota

## Supplementary references

1 Hall, J. K. The 25-m DTM (Digital Terrain Model) of Israel. *Israel Journal of Earth Sciences* **57** (2008).

2 Neugebauer, I. *et al.* Lithology of the long sediment record recovered by the ICDP Dead Sea Deep Drilling Project (DSDDP). *Quaternary Science Reviews* **102**, 149-165 (2014).

3 Kitagawa, H. *et al.* in *Radiocarbon Conference* 1-12 (University of Arizona, Dakar, Senegal, 2016).

4 Reimer, P. J. *et al.* IntCal13 and Marine13 radiocarbon age calibration curves 0–50,000 years cal BP. *Radiocarbon* **55**, 1869-1887 (2013).
